# Maternal nutrition during mid-pregnancy and children’s body composition at 7 years of age in the SELMA study

**DOI:** 10.1017/S0007114523000983

**Published:** 2023-12-14

**Authors:** Katherine Svensson, Chris Gennings, Lars Hagenäs, Alicja Wolk, Niclas Håkansson, Sverre Wikström, Carl-Gustaf Bornehag

**Affiliations:** 1 Department of Health Sciences, Karlstad University, Karlstad, Sweden; 2 Department of Environmental Medicine and Public Health, Icahn School of Medicine at Mount Sinai, New York, USA; 3 Department of Women’s and Children’s Health, Karolinska Institutet, Stockholm, Sweden; 4 Institute of Environmental Medicine, Karolinska Institutet, Stockholm, Sweden; 5 Centre for Clinical Research and Education, County Council of Värmland, Värmland County, Sweden

**Keywords:** Body composition, Nutrition, Pregnancy, Children

## Abstract

Optimal nutrition during pregnancy is vital for both maternal and child health. Our objective was to explore if prenatal diet is associated with children’s height and body fat. Nutrient intake was assessed through a FFQ from 808 pregnant women and summarised to a nutrition index, ‘My Nutrition Index’ (MNI). The association with children’s height and body fat (bioimpedance) was assessed with linear regression models. Secondary analysis was performed with BMI, trunk fat and skinfolds. Overall, higher MNI score was associated with greater height (*β* = 0·47; (95 % CI 0·00, 0·94), among both sexes. Among boys, higher MNI was associated with 0·15 higher BMI z-scores, 0·12 body fat z-scores, 0·11 trunk fat z-scores, and larger triceps, and triceps + subscapular skinfolds (*β* = 0·05 and *β* = 0·06; on the log2 scale) (*P*-value < 0·05). Among girls, the opposite associations were found with 0·12 lower trunk fat z-scores, and smaller subscapular and suprailiac skinfolds (*β* = −0·07 and *β* = −0·10; on the log2 scale) (*P*-value < 0·05). For skinfold measures, this would represent a ± 1·0 millimetres difference. Unexpectedly, a prenatal diet in line with recommended nutrient intake was associated with higher measures of body fat for boys and opposite to girls at a pre-pubertal stage of development.

The hypothesis of Developmental Origins of Health and Disease (DOHaD) states that environmental exposures during pregnancy, including nutrition, may influence growth and development^(1)^. Initial work of David Barker showed that prenatal malnutrition has a long-lasting effect on fetal growth and may result in higher risk for CVD in adulthood^(2,3)^. Famine studies have shown that adults exposed to famine *in utero* have higher odds of glucose intolerance, higher blood pressure and hyperglycaemia later in life^(4–6)^. In addition, animal models with maternal nutrition restriction have smaller offspring, long-standing effects in organ development and epigenetics changes^(7–9)^. The long-term health effects of exposure to famine while *in utero* may also be different for males and females with females portraying more body fat in adulthood than males^(10,11)^. Similarly, pregnant mice fed with high-sugar and -fat diet also showed sex-specific differences in offspring’s gene expression^(12)^. Consequently, sex-specific differences would be important to evaluate in the association between prenatal nutrition and children’s body composition.

The first 1000 d from the time of conception until 2 years of age is a critical time of growth and development, especially as many chronic diseases can be programmed by the nutritional status during this period^(13,14)^. Micronutrients play different roles in the fetal development and therefore, the demand for certain micronutrients such as Fe and folic acid changes during pregnancy^(15)^. Deficiencies of macro- and micronutrients have been associated with unfavourable pregnancy outcomes (e.g. pre-eclampsia) and fetal development (e.g. brain and thyroid)^(16,17)^. In general, women in Europe are able to meet the micronutrient requirements (e.g. vitamins and minerals) through a balanced diet, although there is evidence of vitamin D and Fe deficiencies among pregnant women^(18,19)^. Also in high-income countries such as Sweden, there is need for improvement to meet the recommended intake of fruit and vegetables, dairy products and fish^(20)^, as well as folate, vitamin D and Fe intake from foods during pregnancy^(21)^.

Better adherence to recommended nutritional guidelines during pregnancy has been associated with beneficial effect on birth size^(22–26)^. However, the association with children’s body composition after birth is still unclear. Better maternal nutrition has been associated with less body fat at 6 months of age, but also with no association with body fat at 6 and 12 months of age^(24,27)^. Lower vitamin B_12_ status during pregnancy has also been associated with greater body fat and insulin resistance at 6 years of age^(28,29)^, but with no association with children’s body fat at 9 years of age^(30)^. Specifically, dietary supplements of vitamin A, Fe, Zn and folic acid during pregnancy have been associated with greater height and less skinfolds thickness at the age of 6 to 8 years^(31)^. Certain diets during pregnancy, as pro-inflammatory diet, have been associated with higher BMIz among boys^(32)^, and intake of certain fatty acids has been associated with lower odds of obesity for boys but higher for girls^(33)^.

Subsequently, there is not a clear consensus if optimal prenatal nutrition has an effect on children’s body composition later in childhood. The objective of this study was to explore if prenatal nutrition is associated with children’s height and body fat at 7 years of age among 808 mother–child pairs participating in the Swedish Environmental Longitudinal, Mother and child, Asthma and allergy (SELMA) cohort study. Of specific interest was to evaluate potential sex differences between prenatal nutrition and children’s height and body fat.

## Methods

### Study population

The study population for this analysis include women and their children participating in the SELMA study. Pregnant women were recruited during their first prenatal visit (median of 10 week’s gestation) during the years 2007–2010. A total of twenty-five antenatal care centres in the county of Värmland, Sweden, were part of the recruitment process. Women were informed about the study, and a total of 2582 agreed to participate (39 % recruitment rate)^(34)^. The participants were in general slightly older (32 *v*. 31 years), had a greater prevalence of college or university education (50 % *v*. 36 %) and less likely to smoke (14 % *v*. 19 %) as compared with non-participants^(34)^. A total of 1006 of children were followed up at approximately 7 years of age. For this analysis, we selected mother–child pairs for which there was data available on both prenatal diet at mid-pregnancy and body composition measurements at 7 years of age (*n* 829). There was missing data for the following covariates: smoking, maternal education and maternal height (*n* 21). The final study sample of participants with complete data resulted in 808 mother–child pairs. This study was conducted according to the guidelines laid down in the Declaration of Helsinki, and all procedures involving human subjects/patients were approved by the Regional Ethical Review Board in Uppsala, Sweden (Dnr: 2007/062 and Dnr: 2015/177). All participating women signed written informed consent. Written informed parental consent was obtained, and children received age-adapted information about the study procedure.

### Prenatal diet

Maternal prenatal diet was collected during mid-pregnancy through self-reported FFQ. The data collection protocol for the FFQ has been reported elsewhere and described here briefly^(26)^. Self-administered questionnaires were provided to the participants during the prenatal care visit at week 25 of gestation and sent back to the research team by mail. Women were asked to report their overall food intake during the current pregnancy by selecting the food items they consumed and how often. The questionnaire included a total of ninety-eight food items of different categories of food including meat, fish, poultry, eggs, legumes, potatoes, vegetables, grain products and cereals, fruit and berries, cakes and sweets, and other food items (e.g. salad dressing, coffee cream and extra salt). For each item, there were predefined frequency categories: never/rarely, 1–2 times a month, 1–2, 3–4, 5–6 times a week or 1, 2, 3 or more times a day. There were also open questions on daily or weekly consumption of types of milk, yogurt, bread and bread spread (e.g. butter, margarin). For these open questions, the number of glasses or cups (e.g. milk and coffee), the number of slices of bread and tablespoons for bread spread (e.g. soft cheese) were reported. For each item, the frequency of consumption reported per d or per week was then transformed to daily consumption. The quantity for each food item (i.e. grams per d) was calculated by multiplying the frequency of consumption by an age-specific portion size. The portion size was estimated based on the mean value of the 4 × 7-d weighed food records kept by 213 randomly selected women (total of 5922 d) in a previous study^(35)^. The nutrient intake and total energy were calculated based on food composition values from the Swedish National Food Agency database. The current FFQ is an extended version (with additional food items) of a validated FFQ with good reproducibility after 1 year (intraclass correlations 0·54–0·85) and validity (Spearman’s correlations 0·25–0·77) of nutrient estimates^(36)^.

The nutrient intake was then summarised into an index, ‘My Nutrition Index’ (MNI)^(26)^. This index is calculated to estimate adherence to recommended nutrient intake by the Dietary Guidelines for Americans (2015–2020)^(37)^. Even though diet patterns can vary across countries, the American dietary guidelines are in overall very similar to the guidelines used in Sweden, the Nordic Nutrition Recommendations (NNR 2012)^(26,37,38)^. MNI is an indicator of how close each nutrient intake is to the recommended value based on a subject’s characteristics. It assigns a higher score if the nutrient intake is within the range of the recommended intake and a lower score if it is higher or lower than this optimal range. The following macro- and micronutrients considered were total fat, saturated fat, monounsaturated and polyunsaturated fat, protein, carbohydrates, sugar, dietary fibre, vitamin E as *α*-tocopherol, vitamin C, cholesterol, K, Na, Ca, Mg, Fe, P, Zn, thiamin, riboflavin, niacin, vitamin B_6_, vitamin B_12_, vitamin A, vitamin D, folate and Se. The MNI takes into account individual characteristics, including pregnancy status, age, height, weight, overall physical activity level and smoking which changes the recommended nutrient intake. The MNI ranges from 0 to 100 with a higher score reflecting better adherence to the recommended guidelines and a more adequate intake of nutrients for an individual. A perfect score would be if all the nutrient intakes fall within the recommended range of values^(26)^. Consequently, MNI is a measure of diet quality representing how adequate the nutrient intake was for the participating women during pregnancy.

### Children’s body composition

Children were followed up at 7 years of age at health care centres, and body composition measurements were collected according to standard protocols^(39)^. Height was measured with a stadiometer. Weight and percent body fat were measured with an eight-electrode method bioelectrical impedance analysis (BIA) (TANITA BC-418 MA). The BIA estimates the percent body fat using a constant current source with a high-frequency current (50 kHz, 500 μA) which passes through the body. As body fat is less conducive of electric current than water, it is possible to estimate the percent body fat in overall and in different sections of the body. The body type selected on the instrument was ‘standard male’ or ‘standard female’ according to the instruction manual for the in-built manufacturer’s estimation algorithm of body fat. The BIA TANITA BC-418 has shown good agreement with body fat measures from dual-energy X-ray absorptiometry among 7-year-old children and is, therefore, a valid instrument for epidemiological studies^(40)^. All children were measured before breakfast and after emptying the bladder. Children’s BMI z-score was calculated according to the WHO child growth standards for 5–19 years of age^(41,42)^, and the proportion of overweight children was calculated according to the ISO-BMI cut-off levels^(43)^.

Skinfolds were measured using a caliper instrument with 0·1-mm precision to assess subcutaneous fat at the following sites of the body: triceps, subscapular and suprailiac. For this analysis, we also calculated sum of triceps and subscapular sites which have shown good correlations with body fat measured using dual-energy X-ray absorptiometry among school age children^(44)^. All skinfold sites were measured twice on the right side of the body, and the average was calculated and used for analysis. The study visit also included a modified Tanner staging to assess physical signs of puberty. It included parents self-report of children’s pubic hair and visual assessment by a trained nurse of breast development among the girls. However, it did not include assessment of the testicles for the boys. Based on this assessment, none of the children showed any physical signs of puberty.

### Covariates

Maternal age, weight and parity, and child’s sex were retrieved from the Swedish national birth medical registry. Maternal weight was measured during enrolment at the first prenatal care visit (median of 10 week’s gestation). Other characteristics including maternal education level and smoking status were collected through self-administered questionnaires at the time of enrolment. Maternal height, paternal height and weight were also collected through self-administered questionnaires during mid-pregnancy at 25 weeks of gestation. Maternal and paternal height and weight were used to calculate BMI (kg/m^2^). Level of education was collected in the following categories: primary school (9 years), high school (3–4 years) and college/university (3–5 years) or more. These categories were categorised into two groups: less than college/university *v*. college/university or more. Smoking status during pregnancy (median of 10 week’s gestation) was primarily identified through serum cotinine. Women with levels equal or higher than 0·2 ng/ml were considered passive or active smokers, and those with cotinine levels below 0·2 ng/ml were considered non-smokers. For those women who did not have measured cotinine levels (*n* 111), self-reported smoking status was used. Women who answered ‘Yes’ to current smoking were considered passive/active smokers, and those who answered ‘No’ were considered non-smokers. If anyone in the household currently smoked, then the women were considered passive smokers. Parity was dichotomised as primiparous and multiparous.

Maternal and paternal BMI were considered as cofounders in the main and sensitivity analyses as it has been associated with children’s body composition^(45,46)^. Maternal education and smoking are considered cofounders as they are related to behavioural lifestyles and health outcomes^(47,48)^. Smoking during pregnancy is a risk factor for lower birth weight and, therefore, also a possible cofounder for children’s body composition^(49,50)^. Parity has been associated with birth weight, as nulliparity has been associated with higher risk for lower birth weight^(51)^. Children’s birth weight is not a cofounder as it is considered to be on the biological pathway between prenatal diet and body composition in childhood as it is a proxy for fetal growth^(52)^. To take into account extraneous variation in estimates of energy intake resulting from differences in body size, physical activity and metabolic efficiency, we adjusted for this potential dietary cofounding by including energy intake as a covariate in the regression model^(53,54)^.

### Statistical analysis

We used descriptive statistics to assess central tendency measures for all the variables. Skinfold measures were positively skewed and therefore log2-transformed to approximate normal distribution. To assess the relationship between MNI and children’s height and body fat, we performed simple and multiple linear regression models. We also conducted secondary analysis with BMI, trunk fat and skinfolds. In order to assess meaningful differences, we divided the MNI by its interquartile range (IQR = 15·8) to change the unit to an interquartile range instead of one unit on the original scale (0–100). Children’s body fat measures (e.g. overall and trunk) were centred and scaled for easier interpretation. A separate linear regression model was constructed for each of the body composition measures as the outcome and MNI as the predictor. This analysis approach resulted in ten regression models with the outcomes of children’s height, weight, BMI, BMI z-scores, overall percent body fat, percent trunk fat, and skinfold measures of the triceps, subscapular, and suprailiac sites and the sum of triceps and subscapular sites.

The simple linear regression models were adjusted for total energy intake, and the multiple linear models were additionally adjusted for maternal BMI, education, smoking, and parity. Continuous covariates including maternal energy intake and BMI were centred at the mean. In each model, we tested for interaction between MNI and children’s sex by adding an interaction term MNI * sex. The interaction term was significant in all models except for the model with children’s height as outcome (data not shown). Thereafter, we performed stratified regression models in order to identify sex-specific differences in the association between MNI and children’s height and body fat measures.

In addition, we ran sensitivity analyses excluding the highest and lowest fifth percentile of the data resulting in 726 children with BMI between 14 and 20 (kg/m^2^) to remove any potential influence points on the observed associations. Several of the body composition measures are age-adjusted either at the moment of data collection (i.e. BIA) or at the moment of calculation (i.e. BMI z-scores). However, other measures are not age-adjusted including height, weight, BMI and skinfolds. In our study sample, children’s age ranged between 7·0 and 9·4 years (mean and sd: 7·5 ± 0·3). To take children’s age into account, we adjusted the models of non-age-adjusted measures with children’s age as covariate. Even though children’s age is not considered a cofounder, it may be a precision variable for the outcomes due to children’s continued growth throughout childhood^(55)^. We also explored if paternal BMI had an influence on the observed associations besides maternal BMI. A final sensitivity analysis was performed excluding women who were obese (BMI ≥ 30) as maternal BMI is closely related to the child’s body composition^(56)^ and fat tissue also has endocrine activity which may result in more fat deposits in the fetus^(57)^. In addition, we evaluated influential points on total energy intake and MNI. The total energy intake had a median of 1843 kcal and ranged from 686 to 4576 kcal. Five values were above ± 3sd from the mean on the logscale. For MNI, there were eight values below a score of 20. We ran a sensitivity analysis with total energy capped to ± 3 sd, and using ± 3sd as the minimum and maximum value, and MNI capped to a minimum of 20 as an arbitrary criterion for a low score. We also ran a sensitivity analysis excluding the participants with these values (*n* 9).

## Results

### Descriptive results

The study population was comprised by 808 women and their children. The mean and sd of the MNI score was 61·3 ± 13·1 (Table 1). Women with the highest MNI tertile were slightly older (31·3 *v*. 30·9 years), more likely to have a college education level (74 % *v*. 58 %) and more likely to be non-smokers (94 % *v*. 86 %) as compared with women in the lowest tertile (*P*-value < 0·05) (data not shown). Women were in average 30·8 ± 4·6 years old, and about two-thirds (64·2 %) had a college education or more. Most women were non-smokers (90·5 %), and the mean BMI was 24·5 ± 4·3 kg/m^2^. About half of the women had their first child participating in this study (46·0 %). The maternal characteristics did not vary if the child was a boy or a girl. At the follow-up visit, the mean age of the children was 7·5 ± 0·3 years and the mean weight was 26·6 ± 4·6 kg. Boys were taller than the girls, although the mean BMI of 16·0 ± 2·0 kg/m^2^ did not vary by sex. A total of 15·7 % of the children were overweight or obese according to the ISO-BMI classification^(43)^ with a larger percent of the girls being overweight or obese (18·8 %) as compared with the boys (12·7 %). In terms of body fat, the mean body fat was 22·2 % of which 16·1 % was trunk fat. The skinfold measures had a median (IQR) of 8·8 (4·3) mm for triceps, 5·6 (2·7) mm for subscapular and 5·0 (3·2) for suprailiac sites. There were significant differences in body fat between boys and girls, where girls had more percent body fat (23·3 % *v*. 21·0 %) and larger skinfolds as compared with the boys (*P* < 0·001).

**Table 1.** Sociodemographic characteristics of the study population, *n* 808

	Overall (*n* 808)		Boys (*n* 410)		Girls (*n* 398)		*P* *
Maternal characteristics	Mean	sd	Mean	sd	Mean	sd	
My Nutrition Index (MNI)	61·3	13·1	61·5	13·4	61·1	12·8	0·604
Maternal age (years)	30·8	4·6	30·8	4·4	30·9	4·9	0·835
Maternal BMI (kg/m^2^)	24·5	4·3	24·4	4·0	24·7	4·5	0·452
Maternal education	*n*	%	*n*	%	*n*	%	
Less than college/university	289	35·8	145	35·4	144	36·2	
College/university or more	519	64·2	265	64·6	254	63·8	0·866
Smoking							
Non-smoker	731	90·5	369	90·0	362	91·0	
Passive or active smoker	77	9·5	41	10·0	36	9·0	0·732
Parity							
Primiparous	372	46·0	190	46·3	182	45·7	
Multiparous	436	54·0	220	53·7	216	54·3	0·917

Children’s characteristics	Mean	sd	Mean	sd	Mean	sd	
Age at 7 years visit	7·5	0·3	7·5	0·3	7·5	0·3	0·427
Height (cm)	128·5	5·6	129·4	5·6	127·7	5·5	<0·001
Weight (kg)	26·6	4·6	26·9	4·6	26·3	4·6	0·078
BMI (kg/m^2^)	16·0	2·0	16·0	1·9	16·1	2·1	0·546
Body fat (%)	22·2	3·9	21·0	3·7	23·3	3·7	<0·001
Trunk fat (%)	16·1	3·9	15·1	3·7	17·1	3·9	<0·001
Overweight	*n*	%	*n*	%	*n*	%	
Yes (ISO-BMI ≥ 25)	127	15·7	52	12·7	75	18·8	
No (ISO-BMI < 25)	681	84·3	358	87·3	323	81·2	0·021
Skinfolds (mm)	Median	IQR	Median	IQR	Median	IQR	
Triceps	8·8	4·3	8·2	3·8	9·9	4·5	<0·001
Subscapular	5·6	2·7	5·1	2·0	6·2	3·2	<0·001
Suprailiac	5·0	3·2	4·3	2·3	5·6	3·9	<0·001
Triceps + Subscapular	14·5	6·7	13·2	5·1	16·2	7·4	<0·001

* *P*-value from Student’s *t* test for continuous variables (Mann–Whitney for skewed variables) and *χ* ^2^ test for categorical variables.

### Simple and multiple linear regression models

In simple linear regression models adjusting only for energy intake, higher MNI was associated with greater height (*β* = 0·48 cm; 95 % CI 0·02, 0·94) for all children. After adjusting for energy intake, maternal BMI, education, smoking and parity, higher MNI remained significantly associated with greater height (*β* = 0·47 cm; 95 % CI 0·00, 0·94) for all children (online Supplementary Table S1).

**Fig. 1.**
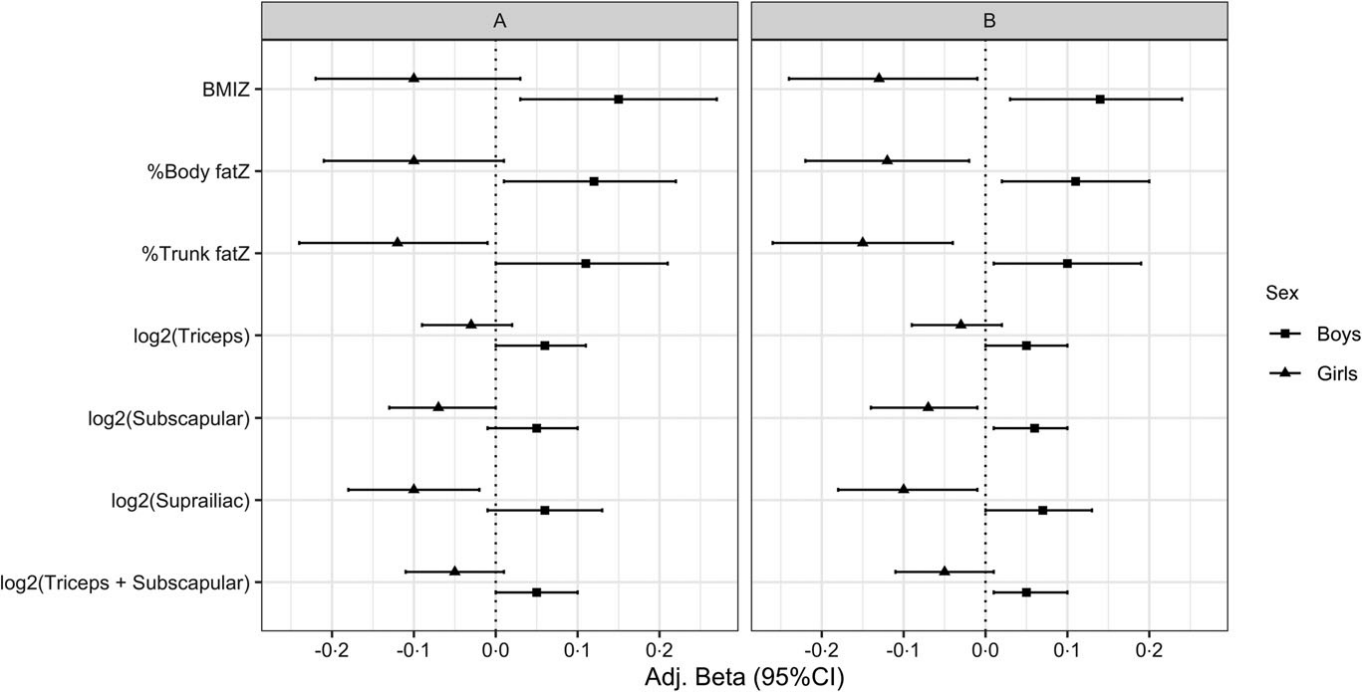
Adjusted associations from linear regression models between prenatal ‘My Nutrition Index’ and children’s body composition stratified for boys and girls, *n* 808. The figure shows adjusted estimates from linear regression models modelling ‘My Nutrition Index’ (MNI) as main predictor and children’s body composition measures as outcome. Results are from stratified models for boys and girls. All models were adjusted for total energy (kcal), maternal BMI, education, smoking and parity. MNI is analysed in units of an interquartile range (IQR = 15·8). Percent body fat (overall and trunk) measures were centred and scaled for easier interpretation. Skinfolds were log2-transformed to approximate normal distribution. (a) All children, *n* 808. (b) Study sample restricted to children with BMI 14–20 (kg/m^2^) by excluding the highest and lowest fifth percentile, *n* 726. The complete results from the models before and after stratifying by sex and including all the selected outcomes (i.e. height) are presented in Supplementary Table S1.

When stratifying by sex, the associations between MNI and children’s body composition were in opposite direction for boys and girls. Among girls, higher MNI score was associated with less trunk fat (*β* = −0·12; 95 % CI −0·25, −0·003) and smaller skinfolds at subscapular (*β* = −0·07; 95 % CI −0·14, −0·002) and suprailiac site (*β* = −0·10; 95 % CI −0·18, −0·02) (estimates on the log2 scale). For boys, the opposite associations were found with higher MNI score associated with greater body fat measures, although these were non-significant. The adjusted estimates remained in the opposite directions for boys and girls even though not all were significant (Fig. 1(a); online Supplementary Table S1). A higher MNI was associated with more trunk fat among boys (*β* = 0·11; 95 % CI 0·002, 0·21) and less among girls (*β* = 0·12; 95 % CI −0·24, −0·01) (standardised score). For boys, there were also significant associations with greater weight (*β* = 0·65; 95 % CI 0·14, 1·16), BMI (*β* = 0·26; 95 % CI 0·05, 0·46), BMI z-scores (*β* = 0·15; 95 % CI 0·03, 0·27), percent body fat (*β* = 0·12; 95 % CI 0·01, 0·22), and triceps skinfolds (*β* = 0·06; 95 % CI 0·004, 0·11) and the sum of triceps and subscapular (*β* = 0·05; 95 % CI 0·004, 0·10). For girls, there were significant associations with smaller subscapular skinfolds (*β* = −0·07; 95 % CI −0·13, −0·003) and suprailiac (*β* = −0·10; 95 % CI −0·18, −0·02). As the skinfolds were log2-transformed to approximate a normal distribution, the estimates represent either an increase for boys or decrease for girls of 1·04–1·05 mm.

### Sensitivity analyses

These linear regression models were also run in a restricted sample removing the children with BMI in the highest and lowest fifth percentile (Fig. 1(b); online Supplementary Table S1). The results were similar as in the models including all children, except MNI was no longer associated with greater height. For boys, the associations remained the same with higher MNI being associated with larger body fat measures. For girls, the previous associations remained significant, and in addition a higher MNI was associated with lower BMI (*β* = −0·24; 95 % CI −0·45, −0·03), BMI z-scores (*β* = −0·13; 95 % CI −0·24, −0·01) and percent body fat (*β* = −0·12; 95 % CI −0·22, −0·02). The complete results from the models before and after stratifying by sex and including all the selected outcomes (i.e. height) are included in Supplementary Table S1.

The sensitivity analyses adjusting for children’s age resulted in similar results. Higher MNI was associated with greater height among all children, although it was no longer significant (*P*-value = 0·063). Distinct opposite associations remained in the estimates among boys and girls for the other body composition measurements (online Supplementary Table S2). Only those models with body measurements which were not age-adjusted during collection or calculation were included in this analysis. The sensitivity analysis adjusting for paternal BMI resulted in the following results. The association between MNI and greater height among all children remained significant. For boys, all estimates were significant with higher MNI being associated with greater weight, BMI, percent body fat and skinfold measurements. However, the associations were no longer significant for girls, although a trend suggested less trunk fat and suprailiac skinfold (*P*-value < 0·08) (online Supplementary Table S3). After excluding mothers with obesity (BMI ≥ 30), the associations (except for weight) remained in opposite directions for boys and girls but were somewhat attenuated. Among boys, higher MNI remained significantly associated with higher BMI (*β* = 0·25; 95 % CI 0·02, 0·48) and BMIz (*β* = 0·15; 95 % CI 0·01, 0·28) and only a trend for higher body fat, subscapular and the sum of triceps and subscapular skinfolds (*P*-value < 0·08). Among girls, the associations were no longer significant (online Supplementary Table S4). The sensitivity analysis with values of total energy capped at ± 3 sd as minimum and maximum value, and MNI capped at 20 as lowest value, did not change our result. Further sensitivity analysis excluding these participants resulted in less significance in the associations, with no significance among girls and a trend for higher body fat among boys (*P*-value = 0·073). However, when restricting to children with BMI 14–20, the results remained the same as in the main analysis.

## Discussion and conclusions

### Maternal nutrition and children’s height

We found that a more nutritious diet during mid-pregnancy, as assessed by the nutrition index MNI, was associated with greater height among 7-year-old children. This is in line with previous studies, including the SELMA study, reporting that better prenatal diet quality has been associated with increased birth size^(22–26)^. Moreover, intake of certain nutrients during pregnancy, specifically Fe and Mg from food sources, has been reported to be associated with increased height at 7·5 years of age^(58)^. Furthermore, malnutrition in pregnancy, as seen in famine studies, is associated with decreased adult height^(59)^. Consequently, enhanced prenatal nutrition may be beneficial for both birth size as well as long-term effect on height.

### Maternal nutrition and children’s body fat

We also found significant sex differences, suggesting that a more nutritious diet is associated with more body fat for boys, but less body fat for girls. A previous study evaluating prenatal diet quality, using the Healthy Eating Index, found an association with lower weight-for-length from birth to 6 months of age, as well as lower body fat at 6 months^(27)^. In contrast, prenatal diet quality assessed by the recommended intake of ten food categories (e.g. fruits and vegetables) found no association with child’s adiposity at birth, 6 and 12 months^(24)^. Studies evaluating specific diet patterns (e.g. Mediterranean diet) or macronutrient (e.g. protein, fat and carbohydrate) intake during pregnancy have found mixed results^(60)^. For example, adhering to a Mediterranean diet during pregnancy has been associated with less body fat in children aged 4 and 7 years as indicated by lower BMIz, waist circumference and skinfolds^(61,62)^. On the other hand, a more ‘unhealthy’ prenatal diet pattern characterised by processed food has been associated with two times the odds of children being overweight at 5 years of age^(63)^. However, there are also studies which have found no association between a ‘healthy’ (e.g. Mediterranean diet) or ‘unhealthy’ (e.g. processed food) diet pattern with the odds of overweight and obesity in children at 6 months, 3 and 5 years of age^(62,64)^. Differences in results may be due to several possibilities. Studies evaluating the association between prenatal diet and children’s body fat have evaluated both specific diet patterns and different indices. To study diet patterns has its advantage from the perspective of health promotion and public health intervention. On the other hand, the advantage of using indices, such as MNI, is using a metric that evaluates the nutritional value of an individual’s diet, irrespective of diet pattern, and how optimal it is compared with the individual’s nutritional need. This may provide a more accurate measure of the nutritional value from foods than specific diet patterns and may be more advantageous from a research perspective. Another aspect that may explain differences across studies is the timing of children’s body fat measures which in previous studies ranges from birth to early- and mid-childhood and also different measures of body fat (e.g. BMI, BIA and skinfolds). These aspects present a challenge when comparing studies. An additional aspect is the importance to evaluate the associations by sex, as there is an indication that the associations may be sex-specific.

### Sex differences in the associations between maternal nutrition and children’s body fat

The association between MNI and body composition was different for boys and girls. In general, there is evidence of metabolic, cardiovascular and anthropometric sex differences in pre-pubertal children^(65–67)^. Also in our study, girls had more body fat as compared with boys which is consistent with previous studies among pre-pubertal children^(66)^. Girls have more peripheral fat (e.g. hip fat) whereas boys have more fat around the waist, and these differences become more distinct during puberty and adulthood^(68)^. However, when it comes to the association between maternal diet and children’s body composition, associations with more body fat were observed among boys and less body fat among girls. There are two previous epidemiological studies showing stratified results by sex. A pro-inflammatory diet, associated with inflammatory markers (e.g. C-reactive protein)^(69,70)^, was associated with higher waist circumference for all children and higher BMIz among boys at 3–5 years of age^(32)^. Also, dietary fatty acid intake during pregnancy had a U-shaped association with odds of obesity. There were differences for boys and girls at 2–7 years of age with certain fatty acids being associated with lower odds of obesity for boys and higher for girls^(33)^. It is difficult to compare our results with these previous two studies as they have evaluated specific prenatal diet patterns. In general, a higher MNI score is an indication of better adherence to the recommended dietary guidelines and reflects a more optimal diet according to the individual dietary needs^(26)^. In that aspect, higher MNI score would most likely promote a healthy environment for fetal growth. In our results, this was associated with more body fat for boys and less for girls at 7 years of age. The effect size of the associations is somewhat small and, therefore, may not be of clinical relevance on an individual basis. However, from an epidemiological perspective, our results add further insight that prenatal nutrition may influence children’s body fat even after birth with sex-specific effects. Consequently, future studies evaluating sex-specific associations are needed in order to replicate and further elucidate these findings.

### Maternal nutrition reflects socio-economic status and lifestyle

Prenatal diet quality also reflects a certain lifestyle or nutritional household environment which may be adopted by the child^(71)^. Prenatal nutrition interacts with socio-economic status (SES) and lifestyle, and healthier dietary pattern has been described among pregnant women who have higher income and education and less likely to be smokers^(24,27,72)^. Adherence to the Nordic recommended dietary guidelines during pregnancy also increases with age, SES and exercise^(73)^. In this regard, higher SES may promote healthier choices as well as provide more possibilities to access healthier options. In Sweden, child overweight and obesity are related to SES and urbanisation level. The odds of overweight is higher in low-educational and rural areas among children of 7–9 years of age^(74)^. This interaction between prenatal nutrition, SES and child’s body composition would be important to evaluate in future studies. Correlation between parental and child’s dietary habits is influenced by family meals and parental practices^(75)^. There is also a correlation between maternal prenatal and postnatal nutrition^(76)^, and this suggests there is a dietary household environment that remains after pregnancy and is inherited by the child^(75)^. In addition, sensitivity analysis involving maternal and paternal BMI resulted in attenuated associations for both boys and girls. This is in line with previous studies showing that parental BMI is associated with children’s body composition and is an indicator of both genetics and lifestyle^(45,56)^. Prenatal diet may therefore be associated with children’s body composition through SES and dietary lifestyle inherited from the parents. However, SES and lifestyle may not explain the observed sex differences in the association between maternal nutrition and children’s body composition.

### Epigenetic changes may explain the sex-specific response

Maternal nutrition has the potential to indirectly impact the metabolism of offspring through epigenetic changes^(77)^. In animal models, protein-restricted diet as well as diet high in carbohydrates have an effect on several cardiometabolic markers in offspring such as insulin sensitivity, lipid metabolism, lean and fat mass and blood pressure^(78–80)^. There is evidence that males and females may respond differently when it comes to epigenetic mechanisms influenced by prenatal diet. In mice, a high-sugar and -fat diet during pregnancy resulted in lower insulin signalling in skeletal muscle tissue in female offspring, whereas males had a lower mitochondrial complex expression^(12)^. Previous research also suggests that prenatal nutrition may influence the placenta in a sex-specific manner^(81)^. This is of importance as the placenta regulates the transport of nutrients and gas to the growing fetus^(82)^. In a famine study, maternal undernutrition was associated with lower placental area, and in greater extent for boys^(83)^. In animal models, it was observed that baboons fed with a restricted diet during pregnancy resulted in upregulated gene expression in the female placentas, whereas the male placenta did not show the same adaptive response^(84)^. Also, a high-fat diet in rabbits was associated with greater lipid storage in the female placenta than the male placenta, and the expression of genes related to lipid pathways was reduced in the male placenta^(85)^. These findings propose there is a sex-specific response in both placental and fetal growth in relation to prenatal diet.

The impact of maternal nutrition on fetal growth and epigenetic programming may be greater in the first trimester. Animal studies show that nutrition restriction in early gestation had an effect on mRNA abundance and adiposity at term, as compared with no effect on nutrition restriction in late gestation^(86)^. Results from the Dutch famine show that exposure to famine in early gestation had a greater effect on the placental size for boys^(83)^, as well as greater risk for CHD in adulthood^(87)^. Women in the SELMA study were asked to provide an average of their diet during the current pregnancy which would represent a proxy for early- and mid-pregnancy. It could be possible that a greater effect would be seen with nutrition limited to only early gestation.

### Strengths and limitations

Our results should be interpreted in view of the study’s strengths and limitations. Strengths of this study include the SELMA study design of a pregnancy cohort with a comprehensive battery of measurements from a large study population. This study design allows to evaluate long-term associations between prenatal diet and children’s body composition. Using MNI as the nutrition index is advantageous as it takes into account an individual’s characteristics when calculating the adherence to the recommended nutritional guidelines. It has also demonstrated to have good predictive validity in well-established health associations with birth weight and cognition^(26)^. Also, on a population level, MNI has demonstrated to have good predictive validity with several population health outcomes (e.g. obesity, depression and CVD)^(88)^. Another strength is that several measures of body fat (e.g. BMI, body fat and skinfolds) were included which increases the reliability of the associations found. BMI is the most widely used indicator for body fat, whereas electrical bioimpedance and skinfolds are considered more reliable measures^(39)^. Also, the body composition measures were performed at the age of 7 years which is a stable time in children’s growth, and it is expected that children are slimmer as compared with early childhood. None of the children participating in the SELMA study had reached puberty at this study visit.

There are also limitations to consider. In terms of diet, a similar FFQ questionnaire to the one applied in the SELMA study has shown good reproducibility and validity of specific nutrients^(36)^. Nevertheless, the FFQ used in this study was not identical with the validated one. The FFQ questionnaire was applied only once during pregnancy and, therefore, we are not able to evaluate changes in diet throughout pregnancy. However, the collected information is an overall representation of the women’s diet during early- and mid-pregnancy and any misclassification due to individual variability with respect to recall bias would only draw the associations towards the null. Our analysis is limited to the evaluation of adherence to the dietary guidelines through food intake only. MNI assesses the nutritional adequacy of an individual’s daily nutrient intake from foods and does not consider intake of dietary supplements which may lead to underestimation of vitamins and mineral intake. However, our goal was not to provide recommendations of pregnant women’s diet or total nutrient intake but rather evaluate if their individual food-based nutrient intake was associated with children’s height and body fat. Additionally, we cannot see a reason why such underestimation of nutrient intake would explain the found sex-specific associations with children’s body fat. The MNI was calculated based on the Dietary Guidelines for Americans (2015–2020) which are similar to NNR 2012. A few differences are worth highlighting, as the NNR recommend less saturated fat and higher potassium intake as compared with the American guidelines^(26)^. However, MNI summarises the adherence to the dietary guidelines in overall and differences in those two individual micronutrients may not be a major effect on the overall scoring. Previous literature suggests that maternal and child nutrition are correlated. Unfortunately, diet intake has not been assessed in the children, and therefore we were not able to include it in our analysis. Even though none of the children had shown signs of puberty, we were not able to evaluate any hormonal changes (e.g. dehydroepiandrosterone sulphate) which may occur before any physical signs of puberty appear. From previous studies, difference in dehydroepiandrosterone sulphate concentrations between boys and girls may explain greater body fat among girls^(66)^. Another limitation is that we do not have information on gestational weight gain (GWG) in the SELMA study as maternal weight at delivery has not consistently been entered in the Swedish national birth medical registry and information is missing for approximately 60 % of the births in the 2010s^(89)^. Although gestational weight gain is a risk factor for adverse pregnancy outcome, recent studies show that obesity before pregnancy may be more of a concern^(90,91)^. Our analysis was adjusted for maternal BMI from early pregnancy (median of 10 week’s gestation), and among the participating women there were 12·4 % who were obese (BMI ≥ 30) and 1·7 % were underweight (BMI < 18·5). The sensitivity analysis excluding obese women showed attenuation of the associations, especially for girls. Our sample was not big enough to explore this further, but future studies may want to look at stratified analyses for women with and without obesity. This is to evaluate the effect of adherence to dietary guidelines among pregnant women with and without obesity and their child’s body composition. Besides maternal weight, a recommendation for future studies would be to measure metabolic markers during pregnancy such as insulin, glucose and lipids for more insight on the metabolic and nutritional uterine environment^(92)^.

### Conclusions

In summary, our study found that enhanced prenatal diet quality was associated with greater height among children at 7 years of age. Better diet quality during pregnancy was, however, unexpectedly associated with more body fat among boys and the opposite for girls. Future studies are warranted to further confirm and evaluate the long-term effects of prenatal diet quality on body fat for boys and girls in childhood, puberty and adulthood.

## Supplementary material

For supplementary material accompanying this paper visit https://doi.org/10.1017/S0007114523000983. click here to view supplementary material

## References

[1] Hoffman DJ , Reynolds RM & Hardy DB (2017) Developmental origins of health and disease: current knowledge and potential mechanisms. Nutr Rev 75, 951–970. 29186623 10.1093/nutrit/nux053

[2] Barker DJ (1997) Fetal nutrition and cardiovascular disease in later life. Br Med Bull 53, 96–108. 9158287 10.1093/oxfordjournals.bmb.a011609

[3] Barker DJ (2007) The origins of the developmental origins theory. J Intern Med 261, 412–417. 17444880 10.1111/j.1365-2796.2007.01809.x

[4] Li Y , He Y , Qi L , et al. (2010) Exposure to the Chinese famine in early life and the risk of hyperglycemia and type 2 diabetes in adulthood. Diabetes 59, 2400–2406. 20622161 10.2337/db10-0385 PMC3279550

[5] Ravelli AC , van der Meulen JH , Michels RP , et al. (1998) Glucose tolerance in adults after prenatal exposure to famine. Lancet 351, 173–177. 9449872 10.1016/s0140-6736(97)07244-9

[6] Stein AD , Zybert PA , van der Pal-de Bruin K , et al. (2006) Exposure to famine during gestation, size at birth, and blood pressure at age 59 years: evidence from the Dutch Famine. Eur J Epidemiol 21, 759–765. 17082900 10.1007/s10654-006-9065-2

[7] Elias AA , Ghaly A , Matushewski B , et al. (2016) Maternal nutrient restriction in Guinea Pigs as an animal model for inducing fetal growth restriction. Reprod Sci 23, 219–227. 26342049 10.1177/1933719115602773

[8] Masoumy EP , Sawyer AA , Sharma S , et al. (2018) The lifelong impact of fetal growth restriction on cardiac development. Pediatr Res 84, 537–544. 29967522 10.1038/s41390-018-0069-x PMC6265071

[9] Nijland MJ , Mitsuya K , Li C , et al. (2010) Epigenetic modification of fetal baboon hepatic phosphoenolpyruvate carboxykinase following exposure to moderately reduced nutrient availability. J Physiol 588, 1349–1359. 20176628 10.1113/jphysiol.2009.184168 PMC2872738

[10] Ravelli AC , Van Der Meulen JH , Osmond C , et al. (1999) Obesity at the age of 50 years in men and women exposed to famine prenatally. Am J Clin Nutr 70, 811–816. 10539740 10.1093/ajcn/70.5.811

[11] Stein AD , Kahn HS , Rundle A , et al. (2007) Anthropometric measures in middle age after exposure to famine during gestation: evidence from the Dutch famine. Am J Clin Nutr 85, 869–876. 17344511 10.1093/ajcn/85.3.869

[12] Shelley P , Martin-Gronert MS , Rowlerson A , et al. (2009) Altered skeletal muscle insulin signaling and mitochondrial complex II-III linked activity in adult offspring of obese mice. Am J Physiol Regul Integr Comp Physiol 297, R675–681. 19535678 10.1152/ajpregu.00146.2009 PMC2739782

[13] Mameli C , Mazzantini S & Zuccotti GV (2016) Nutrition in the first 1000 days: the origin of childhood obesity. Int J Environ Res Public Health 13, 838. 27563917 10.3390/ijerph13090838 PMC5036671

[14] Schwarzenberg SJ & Georgieff MK (2018) Advocacy for improving nutrition in the first 1000 days to support childhood development and adult health. Pediatr 141, e20173716. 10.1542/peds.2017-3716 29358479

[15] Jouanne M , Oddoux S , Noël A , et al. (2021) Nutrient requirements during pregnancy and lactation. Nutrients 13, 692. 33670026 10.3390/nu13020692 PMC7926714

[16] Christian P , Mullany LC , Hurley KM , et al. (2015) Nutrition and maternal, neonatal, and child health. Semin Perinatol 39, 361–372. 26166560 10.1053/j.semperi.2015.06.009

[17] Danielewicz H , Myszczyszyn G , Dębińska A , et al. (2017) Diet in pregnancy-more than food. Eur J Pediatr 176, 1573–1579. 29101450 10.1007/s00431-017-3026-5 PMC5682869

[18] Lips P , Cashman KD , Lamberg-Allardt C , et al. (2019) Current vitamin D status in European and Middle East countries and strategies to prevent vitamin D deficiency: a position statement of the European Calcified Tissue Society. Eur J Endocrinol 180, P23–P54. 30721133 10.1530/EJE-18-0736

[19] Milman N , Taylor CL , Merkel J , et al. (2017) Iron status in pregnant women and women of reproductive age in Europe. Am J Clin Nutr 106, 1655S–1662S. 29070543 10.3945/ajcn.117.156000 PMC5701710

[20] Wennberg AL , Isaksson U , Sandström H , et al. (2016) Swedish women’s food habits during pregnancy up to six months post-partum: a longitudinal study. Sex Reprod Healthc 8, 31–36. 27179375 10.1016/j.srhc.2016.01.006

[21] Lundqvist A , Johansson I , Wennberg A , et al. (2014) Reported dietary intake in early pregnant compared to non-pregnant women – a cross-sectional study. BMC Pregnancy Childbirth 14, 373. 25361589 10.1186/s12884-014-0373-3 PMC4221707

[22] Ancira-Moreno M , O’Neill MS , Rivera-Dommarco J , et al. (2020) Dietary patterns and diet quality during pregnancy and low birthweight: the PRINCESA cohort. Matern Child Nutr 16, e12972. 32037674 10.1111/mcn.12972 PMC7296796

[23] Chen LW , Aubert AM , Shivappa N , et al. (2021) Associations of maternal dietary inflammatory potential and quality with offspring birth outcomes: an individual participant data pooled analysis of 7 European cohorts in the ALPHABET consortium. PLoS Med 18, e1003491. 33476335 10.1371/journal.pmed.1003491 PMC7819611

[24] Gonzalez-Nahm S , Hoyo C , Østbye T , et al. (2019) Associations of maternal diet with infant adiposity at birth, 6 months and 12 months. BMJ Open 9, e030186. 10.1136/bmjopen-2019-030186 PMC6731802 31494614

[25] Yisahak SF , Mumford SL , Grewal J , et al. (2021) Maternal diet patterns during early pregnancy in relation to neonatal outcomes. Am J Clin Nutr 114, 358–367. 33742192 10.1093/ajcn/nqab019 PMC8246623

[26] Gennings C , Wolk A , Hakansson N , et al. (2020) Contrasting prenatal nutrition and environmental exposures in association with birth weight and cognitive function in children at 7 years. BMJ Nutr Prev Health 3, 162–171. 10.1136/bmjnph-2020-000099 PMC7841844 33521525

[27] Tahir MJ , Haapala JL , Foster LP , et al. (2019) Higher maternal diet quality during pregnancy and lactation is associated with lower infant weight-for-length, body fat percent, and fat mass in early postnatal life. Nutrients 11, 632. 30875943 10.3390/nu11030632 PMC6471184

[28] Yajnik CS , Deshpande S , Jackson A , et al. (2008) Vitamin B_12_ and folate concentrations during pregnancy and insulin resistance in the offspring: the Pune Maternal Nutrition Study. Diabetologia 51, 29–38. 17851649 10.1007/s00125-007-0793-y PMC2100429

[29] Stewart CP , Christian P , Schulze KJ , et al. (2011) Low maternal vitamin B-12 status is associated with offspring insulin resistance regardless of antenatal micronutrient supplementation in rural Nepal. J Nutr 141, 1912–1917. 21865563 10.3945/jn.111.144717

[30] Lewis SJ , Leary S , Davey Smith G , et al. (2009) Body composition at age 9 years, maternal folate intake during pregnancy and methyltetrahydrofolate reductase (MTHFR) C677T genotype. Br J Nutr 102, 493–496. 19660149 10.1017/S0007114509231746

[31] Stewart CP , Christian P , LeClerq SC , et al. (2009) Antenatal supplementation with folic acid + iron + zinc improves linear growth and reduces peripheral adiposity in school-age children in rural Nepal. Am J Clin Nutr 90, 132–140. 19474130 10.3945/ajcn.2008.27368 PMC2696997

[32] Sen S , Rifas-Shiman SL , Shivappa N , et al. (2018) Associations of prenatal and early life dietary inflammatory potential with childhood adiposity and cardiometabolic risk in Project Viva. Pediatr Obes 13, 292–300. 28493362 10.1111/ijpo.12221 PMC5681442

[33] Hakola L , Takkinen HM , Niinistö S , et al. (2017) Maternal fatty acid intake during pregnancy and the development of childhood overweight: a birth cohort study. Pediatr Obes 12, 26–37. 27378525 10.1111/ijpo.12170

[34] Bornehag CG , Moniruzzaman S , Larsson M , et al. (2012) The SELMA study: a birth cohort study in Sweden following more than 2000 mother-child pairs. Paediatr Perinat Epidemiol 26, 456–467. 22882790 10.1111/j.1365-3016.2012.01314.x

[35] Khani BR , Ye W , Terry P , et al. (2004) Reproducibility and validity of major dietary patterns among Swedish women assessed with a food-frequency questionnaire. J Nutr 134, 1541–1545. 15173426 10.1093/jn/134.6.1541

[36] Messerer M , Johansson SE & Wolk A (2004) The validity of questionnaire-based micronutrient intake estimates is increased by including dietary supplement use in Swedish men. J Nutr 134, 1800–1805. 15226472 10.1093/jn/134.7.1800

[37] U.S. Department of Health and Human Services and U.S. Department of Agriculture (2015) 2015–2020 Dietary Guidelines for Americans. http://health.gov/dietaryguidelines/2015/guidelines/ (accessed December, 2022).

[38] Ministerråd N (2013) Nordic Nutrition Recommendations 2012. Part 1: Summary, Principles and Use. Copenhagen: Nordic Council of Ministers.

[39] Jensen N , Camargo T & Bergamaschi D (2016) Comparison of methods to measure body fat in 7-to-10-year-old children: a systematic review. Public Health 133, 3–13. 26774698 10.1016/j.puhe.2015.11.025

[40] Luque V , Closa-Monasterolo R , Rubio-Torrents C , et al. (2014) Bioimpedance in 7-year-old children: validation by dual X-ray absorptiometry - part 1: assessment of whole body composition. Ann Nutr Metab 64, 113–121. 24992892 10.1159/000356450

[41] WHO Multicentre Growth Reference Study Group & de Onis M (2006) WHO Child Growth Standards based on length/height, weight and age. Acta Paediatr 95, 76–85. 10.1111/j.1651-2227.2006.tb02378.x 16817681

[42] de Onis M , Onyango AW , Borghi E , et al. (2007) Development of a WHO growth reference for school-aged children and adolescents. Bull World Health Organ 85, 660–667. 18026621 10.2471/BLT.07.043497 PMC2636412

[43] Cole TJ , Bellizzi MC , Flegal KM , et al. (2000) Establishing a standard definition for child overweight and obesity worldwide: international survey. BMJ 320, 1240–1243. 10797032 10.1136/bmj.320.7244.1240 PMC27365

[44] Boeke CE , Oken E , Kleinman KP , et al. (2013) Correlations among adiposity measures in school-aged children. BMC Pediatr 13, 99. 23799991 10.1186/1471-2431-13-99 PMC3693882

[45] Patro B , Liber A , Zalewski B , et al. (2013) Maternal and paternal body mass index and offspring obesity: a systematic review. Ann Nutr Metab 63, 32–41. 23887153 10.1159/000350313

[46] Svensson V , Jacobsson JA , Fredriksson R , et al. (2011) Associations between severity of obesity in childhood and adolescence, obesity onset and parental BMI: a longitudinal cohort study. Int J Obes 35, 46–52. 10.1038/ijo.2010.189 PMC3035977 20856258

[47] Johansson SE & Sundquist J (1999) Change in lifestyle factors and their influence on health status and all-cause mortality. Int J Epidemiol 28, 1073–1080. 10661650 10.1093/ije/28.6.1073

[48] Benzies KM , Wångby M & Bergman LR (2008) Stability and change in health-related behaviors of midlife Swedish women. Health Care Women Int 29, 997–1018. 18821211 10.1080/07399330802269675

[49] Pereira PP , Da Mata FA , Figueiredo AC , et al. (2017) Maternal active smoking during pregnancy and low birth weight in the Americas: a systematic review and meta-analysis. Nicotine Tobacco Res 19, 497–505. 10.1093/ntr/ntw228 28403455

[50] Zaren B , Lindmark G & Gebre-Medhin M (1996) Maternal smoking and body composition of the newborn. Acta Paediatrica 85, 213–219. 8640053 10.1111/j.1651-2227.1996.tb13995.x

[51] Shah PS & Births KSGoDoLP (2010) Parity and low birth weight and preterm birth: a systematic review and meta-analyses. Acta Obstet Gynecol Scand 89, 862–875. 20583931 10.3109/00016349.2010.486827

[52] Oken E & Gillman MW (2003) Fetal origins of obesity. Obes Res 11, 496–506. 12690076 10.1038/oby.2003.69

[53] McCullough LE & Byrd DA (2022) Total energy intake: implications for epidemiologic analyses. Am J Epidemiol. kwac071. 10.1093/aje/kwac071 35419586

[54] Willett WC , Howe GR & Kushi LH (1997) Adjustment for total energy intake in epidemiologic studies. Am J Clin Nutr 65, 1220S–1228S. 9094926 10.1093/ajcn/65.4.1220S

[55] Schisterman EF , Cole SR & Platt RW (2009) Overadjustment bias and unnecessary adjustment in epidemiologic studies. Epidemiology 20, 488. 19525685 10.1097/EDE.0b013e3181a819a1 PMC2744485

[56] Whitaker KL , Jarvis MJ , Beeken RJ , et al. (2010) Comparing maternal and paternal intergenerational transmission of obesity risk in a large population-based sample. Am J Clin Nutr 91, 1560–1567. 20375189 10.3945/ajcn.2009.28838

[57] Desoye G & Herrera E (2021) Adipose tissue development and lipid metabolism in the human fetus: the 2020 perspective focusing on maternal diabetes and obesity. Prog Lipid Res 81, 101082. 33383022 10.1016/j.plipres.2020.101082

[58] Leary S , Ness A , Emmett P , et al. (2005) Maternal diet in pregnancy and offspring height, sitting height, and leg length. J Epidemiol Community Health 59, 467. 15911641 10.1136/jech.2004.029884 PMC1757047

[59] Arage G , Belachew T & Abate KH (2022) Early life famine exposure and anthropometric profile in adulthood: a systematic review and meta-analysis. BMC Nutr 8, 36. 35459231 10.1186/s40795-022-00523-w PMC9028079

[60] Litvak J , Parekh N & Deierlein A (2020) Prenatal dietary exposures and offspring body size from 6 months to 18 years: a systematic review. Paediatric Perinatal Epidemiol 34, 171–189. 10.1111/ppe.12629 32011754

[61] Chatzi L , Rifas-Shiman SL , Georgiou V , et al. (2017) Adherence to the Mediterranean diet during pregnancy and offspring adiposity and cardiometabolic traits in childhood. Pediatr Obes 12, 47–56. 28160450 10.1111/ijpo.12191 PMC5697744

[62] Fernández-Barrés S , Romaguera D , Valvi D , et al. (2016) Mediterranean dietary pattern in pregnant women and offspring risk of overweight and abdominal obesity in early childhood: the INMA birth cohort study. Pediatr Obes 11, 491–499. 26763767 10.1111/ijpo.12092

[63] Murrin CM , Heinen MM & Kelleher CC (2015) Are dietary patterns of mothers during pregnancy related to children’s weight status? Evidence from the Lifeways Cross- Generational Cohort Study. AIMS Public Health 2, 274–296. 29546111 10.3934/publichealth.2015.3.274 PMC5690236

[64] Martin CL , Siega-Riz AM , Sotres-Alvarez D , et al. (2016) Maternal dietary patterns during pregnancy are associated with child growth in the first 3 years of life. J Nutr 146, 2281–2288. 27683873 10.3945/jn.116.234336 PMC5086788

[65] Arfai K , Pitukcheewanont PD , Goran MI , et al. (2002) Bone, muscle, and fat: sex-related differences in prepubertal children. Radiol 224, 338–344. 10.1148/radiol.2242011369 12147825

[66] Ayyavoo A , Derraik JG , Hofman PL , et al. (2014) Metabolic, cardiovascular and anthropometric differences between prepubertal girls and boys. Clin Endocrinol 81, 238–243. 10.1111/cen.12436 24612121

[67] Taylor RW , Gold E , Manning P , et al. (1997) Gender differences in body fat content are present well before puberty. Int J Obes Relat Metab Disord 21, 1082–1084. 9368835 10.1038/sj.ijo.0800522

[68] Taylor RW , Grant AM , Williams SM , et al. (2010) Sex differences in regional body fat distribution from pre- to postpuberty. Obesity 18, 1410–1416. 19893501 10.1038/oby.2009.399

[69] Shivappa N , Steck SE , Hurley TG , et al. (2014) Designing and developing a literature-derived, population-based dietary inflammatory index. Public Health Nutr 17, 1689–1696. 23941862 10.1017/S1368980013002115 PMC3925198

[70] Sen S , Rifas-Shiman SL , Shivappa N , et al. (2016) Dietary inflammatory potential during pregnancy is associated with lower fetal growth and breastfeeding failure: results from project viva. J Nutr 146, 728–736. 26936137 10.3945/jn.115.225581 PMC4807648

[71] Shrivastava A , Murrin C , Sweeney MR , et al. (2013) Familial intergenerational and maternal aggregation patterns in nutrient intakes in the Lifeways Cross-Generation Cohort Study. Public Health Nutr 16, 1476–1486. 22883601 10.1017/S1368980012003667 PMC10271879

[72] Caut C , Leach M & Steel A (2020) Dietary guideline adherence during preconception and pregnancy: a systematic review. Matern Child Nutr 16, e12916. 31793249 10.1111/mcn.12916 PMC7083492

[73] von Ruesten A , Brantsæter AL , Haugen M , et al. (2014) Adherence of pregnant women to Nordic dietary guidelines in relation to postpartum weight retention: results from the Norwegian Mother and Child Cohort Study. BMC Public Health 14, 75. 24456804 10.1186/1471-2458-14-75 PMC3908932

[74] Sjöberg A , Moraeus L , Yngve A , et al. (2011) Overweight and obesity in a representative sample of schoolchildren - exploring the urban-rural gradient in Sweden. Obes Rev 12, 305–314. 21348925 10.1111/j.1467-789X.2010.00838.x

[75] Mahmood L , Flores-Barrantes P , Moreno LA , et al. (2021) The influence of parental dietary behaviors and practices on children’s eating habits. Nutrients 13, 1138. 33808337 10.3390/nu13041138 PMC8067332

[76] Crozier SR , Robinson SM , Godfrey KM , et al. (2009) Women’s dietary patterns change little from before to during pregnancy. J Nutr 139, 1956–1963. 19710161 10.3945/jn.109.109579 PMC3113465

[77] Tobi EW , Goeman JJ , Monajemi R , et al. (2014) DNA methylation signatures link prenatal famine exposure to growth and metabolism. Nat Commun 5, 5592. 25424739 10.1038/ncomms6592 PMC4246417

[78] Kereliuk SM , Brawerman GM & Dolinsky VW (2017) Maternal macronutrient consumption and the developmental origins of metabolic disease in the offspring. Int J Mol Sci 18, 1451. 28684678 10.3390/ijms18071451 PMC5535942

[79] Borengasser SJ , Zhong Y , Kang P , et al. (2013) Maternal obesity enhances white adipose tissue differentiation and alters genome-scale DNA methylation in male rat offspring. Endocrinol 154, 4113–4125. 10.1210/en.2012-2255 PMC3800750 23959936

[80] Clayton ZE , Vickers MH , Bernal A , et al. (2015) Early life exposure to fructose alters maternal, fetal and neonatal hepatic gene expression and leads to sex-dependent changes in lipid metabolism in rat offspring. PLoS One 10, e0141962. 26562417 10.1371/journal.pone.0141962 PMC4643022

[81] Tarrade A , Panchenko P , Junien C , et al. (2015) Placental contribution to nutritional programming of health and diseases: epigenetics and sexual dimorphism. J Exp Biol 218, 50–58. 25568451 10.1242/jeb.110320

[82] Fernandez-Twinn DS & Ozanne SE (2010) Early life nutrition and metabolic programming. Ann NY Acad Sci 1212, 78–96. 21070247 10.1111/j.1749-6632.2010.05798.x

[83] Roseboom TJ , Painter RC , de Rooij SR , et al. (2011) Effects of famine on placental size and efficiency. Placenta 32, 395–399. 21435715 10.1016/j.placenta.2011.03.001

[84] Cox LA , Li C , Glenn JP , et al. (2013) Expression of the placental transcriptome in maternal nutrient reduction in baboons is dependent on fetal sex. J Nutr 143, 1698–1708. 24047701 10.3945/jn.112.172148 PMC3796342

[85] Tarrade A , Rousseau-Ralliard D , Aubrière MC , et al. (2013) Sexual dimorphism of the feto-placental phenotype in response to a high fat and control maternal diets in a rabbit model. PLoS One 8, e83458. 24386205 10.1371/journal.pone.0083458 PMC3873307

[86] Symonds ME , Pearce S , Bispham J , et al. (2004) Timing of nutrient restriction and programming of fetal adipose tissue development. Proc Nutr Soc 63, 397–403. 10.1079/pns2004366 15373949

[87] Roseboom TJ , van der Meulen JHP , Osmond C , et al. (2000) Coronary heart disease after prenatal exposure to the Dutch famine, 1944–1945. Heart 84, 595. 11083734 10.1136/heart.84.6.595 PMC1729504

[88] Busgang SA , Malin AJ & Gennings C (2022) My nutrition index: a method for measuring optimal daily nutrient intake. BMC Nutr 8, 16. 35189956 10.1186/s40795-022-00497-9 PMC8862522

[89] Swedish National Board of Health and Wellfare (2022) The Swedish Birth Medical Registry. https://www.socialstyrelsen.se/en/statistics-and-data/registers/national-medical-birth-register/ (accessed September 2022).

[90] Nohr EA , Vaeth M , Baker JL , et al. (2008) Combined associations of prepregnancy body mass index and gestational weight gain with the outcome of pregnancy. Am J Clin Nutr 87, 1750–1759. 18541565 10.1093/ajcn/87.6.1750

[91] Rasmussen KM , Abrams B , Bodnar LM , et al. (2010) Recommendations for weight gain during pregnancy in the context of the obesity epidemic. Obstet Gynecol 116, 1191–1195. 20966705 10.1097/AOG.0b013e3181f60da7 PMC4288953

[92] Lain KY & Catalano PM (2007) Metabolic changes in pregnancy. Clin Obstet Gynecol 50, 938–948. 17982337 10.1097/GRF.0b013e31815a5494

