# Supplementary material for: Maternal nutrition during mid-pregnancy and children’s body composition at 7 years of age in the SELMA study
Source: Br J Nutr. 2023 May 26;130(11):1982–92. doi: 10.1017/S0007114523000983 (PMC10632724; doi:10.1017/S0007114523000983)
Supplement: Supplementary file 1 [file S0007114523000983sup001.docx]

**Title: Maternal nutrition during mid-pregnancy and children’s body composition at seven years of age in the SELMA study**

Katherine Svensson^1^, Chris Gennings^2^, Lars Hagenäs^3^, Alicja Wolk^4^, Niclas Håkansson^4^, Sverre Wikström^1,5^, Carl-Gustaf Bornehag^1,2^

^1^ Department of Health Sciences, Karlstad University, Karlstad, Sweden

^2^ Department of Environmental Medicine and Public Health, Icahn School of Medicine at Mount Sinai, New York, US

^3^ Department of Women's and Children's Health, Karolinska Institutet, Stockholm, Sweden.

^4^ Institute of Environmental Medicine, Karolinska Institutet, Stockholm, Sweden

^5^ Centre for Clinical Research and Education, County Council of Värmland, Sweden

**Corresponding author:**

Carl-Gustaf Bornehag, PhD

Telephone number: +46 70 586 6565

Email: carl-gustaf.bornehag@kau.se

Address: Department of Health Sciences, Karlstad University, Universitetsgatan 2, 651 88 Karlstad, Sweden

**Supplementary tables**

**Table S1. Adjusted*^a^* associations from linear regression models between prenatal “My Nutrition Index” and children’s body composition, n=808**

|  | **Overall** | | | | **Boys** | | | | **Girls** | | | |
| --- | --- | --- | --- | --- | --- | --- | --- | --- | --- | --- | --- | --- |
|  | **n=808** | | | | **n=410** | | | | **n=398** | | | |
| **Models:** | **Beta** | **(95%CI)** | | **P-value** | **Beta** | **(95%CI)** | | **P-value** | **Beta** | **(95%CI)** | | **P-value** |
| 1. Height (cm) | 0.47 | (0.00, | 0.94) | 0.050 | 0.52 | (-0.12, | 1.16) | 0.114 | 0.35 | (-0.32, | 1.02) | 0.305 |
| 1. Weight (kg) | 0.25 | (-0.12, | 0.63) | 0.187 | 0.65 | (0.14, | 1.16) | 0.014 | -0.19 | (-0.74, | 0.35) | 0.486 |
| 1. BMI (kg/m^2^) | 0.04 | (-0.12, | 0.19) | 0.658 | 0.26 | (0.05, | 0.46) | 0.016 | -0.20 | (-0.44, | 0.04) | 0.102 |
| 1. BMI-z | 0.03 | (-0.06, | 0.12) | 0.477 | 0.15 | (0.03, | 0.27) | 0.015 | -0.10 | (-0.22, | 0.03) | 0.125 |
| 1. Body fat-z (%) | 0.01 | (-0.07, | 0.09) | 0.864 | 0.12 | (0.01, | 0.22) | 0.033 | -0.10 | (-0.21, | 0.01) | 0.069 |
| 1. Trunk fat-z (%) | -0.01 | (-0.09, | 0.07) | 0.868 | 0.11 | (0.002, | 0.21) | 0.047 | -0.12 | (-0.24, | -0.01) | 0.037 |
| Log_2_ (Skinfolds (mm)) |  |  |  |  |  |  |  |  |  |  |  |  |
| 1. *Triceps* | 0.01 | (-0.03, | 0.05) | 0.594 | 0.06 | (0.004, | 0.11) | 0.037 | -0.03 | (-0.09, | 0.02) | 0.256 |
| 1. *Subscapular* | -0.01 | (-0.05, | 0.03) | 0.650 | 0.05 | (-0.01, | 0.10) | 0.088 | -0.07 | (-0.13, | -0.003) | 0.042 |
| 1. *Suprailiac* | -0.02 | (-0.07, | 0.04) | 0.546 | 0.06 | (-0.01, | 0.13) | 0.081 | -0.10 | (-0.18, | -0.02) | 0.017 |
| 1. *Triceps + Subscapular* | 0.002 | (-0.04, | 0.04) | 0.902 | 0.05 | (0.004, | 0.10) | 0.036 | -0.05 | (-0.11, | 0.01) | 0.105 |
| **Restricted to children with BMI index 14-20 (kg/m^2^), n=726** | | | | | | | | | | | | |
|  | **Overall** | | | | **Boys** | | | | **Girls** | | | |
|  | **n=726** | | | | **n=373** | | | | **n=353** | | | |
| **Models:** | **Beta** | **(95%CI)** | | **P-value** | **Beta** | **(95%CI)** | | **P-value** | **Beta** | **(95%CI)** | | **P-value** |
| 1. Height(cm) | 0.28 | (-0.23, | 0.78) | 0.280 | 0.35 | (-0.31, | 1.01) | 0.299 | 0.19 | (-0.57, | 0.94) | 0.625 |
| 1. Weight (kg) | 0.12 | (-0.22, | 0.46) | 0.488 | 0.51 | (0.07, | 0.94) | 0.023 | -0.33 | (-0.85, | 0.20) | 0.221 |
| 1. BMI (kg/m^2^) | 0.01 | (-0.12, | 0.14) | 0.915 | 0.22 | (0.06, | 0.38) | 0.008 | -0.24 | (-0.45, | -0.03) | 0.028 |
| 1. BMI-z | 0.01 | (-0.06, | 0.09) | 0.724 | 0.14 | (0.03, | 0.24) | 0.010 | -0.13 | (-0.24, | -0.01) | 0.027 |
| 1. Body fat-z (%) | 0.004 | (-0.07, | 0.08) | 0.922 | 0.11 | (0.02, | 0.20) | 0.020 | -0.12 | (-0.22, | -0.02) | 0.023 |
| 1. Trunk fat-z (%) | -0.01 | (-0.09, | 0.06) | 0.752 | 0.10 | (0.01, | 0.19) | 0.036 | -0.15 | (-0.26, | -0.04) | 0.010 |
| Log_2_ (Skinfolds (mm)) |  |  |  |  |  |  |  |  |  |  |  |  |
| 1. *Triceps* | 0.01 | (-0.03, | 0.05) | 0.631 | 0.05 | (0.002, | 0.10) | 0.042 | -0.03 | (-0.09, | 0.02) | 0.236 |
| 1. *Subscapular* | -0.005 | (-0.05, | 0.04) | 0.817 | 0.06 | (0.01, | 0.10) | 0.018 | -0.07 | (-0.14, | -0.01) | 0.028 |
| 1. *Suprailiac* | -0.01 | (-0.06, | 0.04) | 0.732 | 0.07 | (0.002, | 0.13) | 0.045 | -0.10 | (-0.18, | -0.01) | 0.021 |
| 1. *Triceps + Subscapular* | 0.003 | (-0.04, | 0.04) | 0.877 | 0.05 | (0.01, | 0.10) | 0.022 | -0.05 | (-0.11, | 0.01) | 0.076 |

*^a^* All models were adjusted for total energy (kcal), maternal BMI, education, smoking, and parity.

Notes: “My Nutrition Index” was analyzed in units of an interquartile range (IQR=15.8). Percent body fat (overall and trunk) measures were centered and scaled. Skinfolds were log_2_ transformed to approximate normal distribution.

**Table S2. Adjusted*^a^* associations from linear regression models between prenatal “My Nutrition Index” and children’s body composition with additional adjustment for child’s age, n=808**

|  | **Overall** | | | | **Boys** | | | | **Girls** | | | |
| --- | --- | --- | --- | --- | --- | --- | --- | --- | --- | --- | --- | --- |
|  | **n=808** | | | | **n=410** | | | | **n=398** | | | |
| **Models:** | **Beta** | **(95%CI)** | | **P-value** | **Beta** | **(95%CI)** | | **P-value** | **Beta** | **(95%CI)** | | **P-value** |
| 1. Height (cm) | 0.43 | (-0.02, | 0.87) | 0.063 | 0.49 | (-0.11, | 1.09) | 0.112 | 0.30 | (-0.35, | 0.95) | 0.361 |
| 1. Weight (kg) | 0.23 | (-0.14, | 0.59) | 0.228 | 0.63 | (0.14, | 1.12) | 0.013 | -0.22 | (-0.76, | 0.32) | 0.417 |
| 1. BMI (kg/m^2^) | 0.03 | (-0.13, | 0.19) | 0.706 | 0.25 | (0.05, | 0.46) | 0.017 | -0.21 | (-0.44, | 0.03) | 0.094 |
| Log_2_ (Skinfolds (mm)) |  |  |  |  |  |  |  |  |  |  |  |  |
| 1. *Triceps* | 0.01 | (-0.03, | 0.05) | 0.621 | 0.06 | (0.003, | 0.11) | 0.039 | -0.03 | (-0.09, | 0.02) | 0.239 |
| 1. *Subscapular* | -0.01 | (-0.06, | 0.03) | 0.601 | 0.05 | (-0.01, | 0.10) | 0.092 | -0.07 | (-0.14, | -0.01) | 0.035 |
| 1. *Suprailiac* | -0.02 | (-0.07, | 0.04) | 0.504 | 0.06 | (-0.01, | 0.13) | 0.085 | -0.10 | (-0.18, | -0.02) | 0.014 |
| 1. *Triceps + Subscapular* | 0.001 | (-0.04, | 0.04) | 0.946 | 0.05 | (0.003, | 0.10) | 0.038 | -0.05 | (-0.11, | 0.01) | 0.093 |

*^a^* All models were adjusted for total energy (kcal), maternal BMI, education, smoking, parity, **and child’s age.**

Notes: “My Nutrition Index” was analyzed in units of an interquartile range (IQR=15.8). Percent body fat (overall and trunk) measures were centered and scaled. Skinfolds were log_2_ transformed to approximate normal distribution.

**Table S3. Adjusted*^a^* associations from linear regression models between prenatal “My Nutrition Index” and children’s body composition with additional adjustment for paternal BMI, n=747**

|  | **Overall** | | | | **Boys** | | | | **Girls** | | | |
| --- | --- | --- | --- | --- | --- | --- | --- | --- | --- | --- | --- | --- |
|  | **n=747** | | | | **n=382** | | | | **n=365** | | | |
| **Outcome:** | **Beta** | **(95%CI)** | | **P-value** | **Beta** | **(95%CI)** | | **P-value** | **Beta** | **(95%CI)** | | **P-value** |
| 1. Height (cm) | 0.53 | (0.03, | 1.02) | 0.037 | 0.47 | (-0.22, | 1.15) | 0.182 | 0.46 | (-0.23, | 1.15) | 0.196 |
| 1. Weight (kg) | 0.35 | (-0.04, | 0.73) | 0.081 | 0.66 | (0.12, | 1.20) | 0.016 | -0.06 | (-0.62, | 0.49) | 0.823 |
| 1. BMI (kg/m^2^) | 0.08 | (-0.08, | 0.24) | 0.342 | 0.28 | (0.07, | 0.49) | 0.010 | -0.15 | (-0.39, | 0.10) | 0.236 |
| 1. BMI-z | 0.05 | (-0.04, | 0.14) | 0.242 | 0.16 | (0.04, | 0.29) | 0.011 | -0.07 | (-0.19, | 0.06) | 0.285 |
| 1. Body fat-z (%) | 0.01 | (-0.07, | 0.10) | 0.747 | 0.13 | (0.02, | 0.24) | 0.019 | -0.08 | (-0.20, | 0.03) | 0.143 |
| 1. Trunk fat-z (%) | -0.001 | (-0.08, | 0.08) | 0.984 | 0.12 | (0.01, | 0.23) | 0.026 | -0.11 | (-0.23, | 0.01) | 0.077 |
| Log_2_ (Skinfolds (mm)) |  |  |  |  |  |  |  |  |  |  |  |  |
| 1. *Triceps* | 0.01 | (-0.03, | 0.06) | 0.523 | 0.06 | (0.01, | 0.11) | 0.033 | -0.02 | (-0.08, | 0.04) | 0.452 |
| 1. *Subscapular* | 0.003 | (-0.04, | 0.05) | 0.898 | 0.07 | (0.01, | 0.12) | 0.021 | -0.05 | (-0.12, | 0.02) | 0.128 |
| 1. *Suprailiac* | -0.003 | (-0.06, | 0.05) | 0.905 | 0.08 | (0.01, | 0.15) | 0.027 | -0.08 | (-0.16, | 0.004) | 0.065 |
| 1. *Triceps + Subscapular* | 0.01 | (-0.03, | 0.05) | 0.636 | 0.06 | (0.01, | 0.11) | 0.016 | -0.03 | (-0.09, | 0.02) | 0.254 |

*^a^* All models were adjusted for total energy (kcal), maternal BMI, education, smoking, parity, **and paternal BMI.**

Notes: “My Nutrition Index” was analyzed in units of an interquartile range (IQR=15.8). Percent body fat (overall and trunk) measures were centered and scaled. Skinfolds were log_2_ transformed to approximate normal distribution.

**Table S4. Adjusted*^a^* associations from linear regression models between prenatal “My Nutrition Index”** **and children’s body composition restricted to maternal BMI<30, n=713**

|  | **Overall** | | | | **Boys** | | | | **Girls** | | | |
| --- | --- | --- | --- | --- | --- | --- | --- | --- | --- | --- | --- | --- |
|  | **n=713** | | | | **n=370** | | | | **n=343** | | | |
| **Outcome:** | **Beta** | **(95%CI)** | | **P-value** | **Beta** | **(95%CI)** | | **P-value** | **Beta** | **(95%CI)** | | **P-value** |
| 1. Height (cm) | 0.58 | (0.08, | 1.07) | 0.023 | 0.56 | (-0.12, | 1.24) | 0.110 | 0.53 | (-0.18, | 1.24) | 0.147 |
| 1. Weight (kg) | 0.36 | (-0.04, | 0.77) | 0.074 | 0.65 | (0.09, | 1.21) | 0.023 | 0.06 | (-0.52, | 0.63) | 0.849 |
| 1. BMI (kg/m^2^) | 0.08 | (-0.09, | 0.25) | 0.364 | 0.25 | (0.02, | 0.48) | 0.035 | -0.09 | (-0.34, | 0.16) | 0.483 |
| 1. BMI-z | 0.05 | (-0.04, | 0.15) | 0.261 | 0.15 | (0.01, | 0.28) | 0.036 | -0.03 | (-0.17, | 0.10) | 0.615 |
| 1. Body fat-z (%) | 0.01 | (-0.07, | 0.10) | 0.776 | 0.10 | (-0.01, | 0.22) | 0.080 | -0.06 | (-0.18, | 0.05) | 0.299 |
| 1. Trunk fat-z (%) | -0.004 | (-0.09, | 0.08) | 0.927 | 0.09 | (-0.02, | 0.21) | 0.113 | -0.09 | (-0.21, | 0.04) | 0.170 |
| Log_2_ (Skinfolds (mm)) |  |  |  |  |  |  |  |  |  |  |  |  |
| 1. *Triceps* | 0.01 | (-0.03, | 0.06) | 0.583 | 0.05 | (-0.01, | 0.11) | 0.110 | -0.01 | (-0.08, | 0.05) | 0.667 |
| 1. *Subscapular* | 0.002 | (-0.05, | 0.05) | 0.920 | 0.06 | (-0.005, | 0.12) | 0.072 | -0.04 | (-0.11, | 0.03) | 0.227 |
| 1. *Suprailiac* | -0.01 | (-0.07, | 0.05) | 0.781 | 0.06 | (-0.02, | 0.14) | 0.118 | -0.07 | (-0.16, | 0.02) | 0.110 |
| 1. *Triceps + Subscapular* | 0.01 | (-0.03, | 0.05) | 0.695 | 0.05 | (-0.004, | 0.11) | 0.069 | -0.03 | (-0.09, | 0.04) | 0.422 |

*^a^* All models were adjusted for total energy (kcal), maternal BMI, education, smoking, parity.

Notes: “My Nutrition Index” was analyzed in units of an interquartile range (IQR=15.8). Percent body fat (overall and trunk) measures were centered and scaled. Skinfolds were log_2_ transformed to approximate normal distribution.
